# Exploring patient perspectives of exercise in advanced pancreatic cancer care in Canada: A qualitative interpretive description

**DOI:** 10.1017/S1478951526103356

**Published:** 2026-09-04

**Authors:** Chloe Cyr, Ben Perry, Ravi Ramjeesingh, Chris Blanchard, Melanie Keats

**Affiliations:** 1 Faculty of Medicine, Dalhousie University, Halifax, Nova Scotia, Canada; 2 Physical Activity and Cancer Lab, Halifax, Nova Scotia, Canada; 3 Beatrice Hunter Cancer Research Institute, Halifax, Nova Scotia, Canada; 4 Craig’s Cause Pancreatic Cancer Society, Halifax, Nova Scotia, Canada; 5 Department of Medicine, Division of Medical Oncology, Dalhousie University, Halifax, Nova Scotia, Canada; 6 School of Health and Human Performance, Dalhousie University, Halifax, Nova Scotia, Canada

**Keywords:** Pancreatic cancer, exercise oncology, behavior change, advanced disease, qualitative

## Abstract

**Objectives:**

Individuals living with advanced pancreatic cancer (APC) face a poor prognosis and high symptom burden. Although exercise training is an evidence-based approach in improving physical and psychological health outcomes in those living with and beyond a cancer diagnosis, there is limited research exploring its role and integration within advanced pancreatic care. The aim of this study was to explore the exercise experiences and perspectives of individuals living with APC in Canada.

**Methods:**

Using a qualitative interpretive description methodology, we conducted semi-structured interviews with 6 individuals diagnosed with advanced (stage III or IV) exocrine pancreatic cancer from across Canada. Interviews were transcribed verbatim, and transcripts were analyzed using reflexive thematic analysis. Inductive and deductive analysis was conducted using the Behaviour Change Wheel and Theoretical Domains Framework as guiding lenses.

**Results:**

Three themes were identified impacting how individuals living with APC perceive and experience exercise: (1) *Taking control of my journey*; (2) *Building the motivation*; and (3) *If it was a program for me.*

**Significance of results:**

This study highlights aspects of the perception of exercise in pancreatic cancer care among individuals living with advanced disease in Canada. Integration of progressive programming, realistic goal setting, and fostering of a supportive environment were identified as important considerations for the care context.

## Introduction

Pancreatic cancer is the third leading cancer-related cause of death in Canada with a 5-year net survival of 10% (Canadian Cancer Statistics Advisory Committee et al. 2023). Exocrine malignancies comprise most pancreatic cancer cases and are often advanced (i.e., locally advanced or metastatic) at diagnosis (Lai and Ung 2024). Pancreatic cancer is associated with a high symptom burden due to malabsorption, altered glucose homeostasis, and pancreatic exocrine insufficiency (Gilliland et al. 2017). These states contribute to severe cachexia and sarcopenia, which are further associated with physical disability, poor treatment tolerance, and decreased survival (Tan et al. 2009; Ozola Zalite et al. 2015; Hendifar et al. 2018; Basile et al. 2019a, 2019b). Compared to individuals with other cancer types, those with pancreatic cancer experience significantly worse psychological well-being (Clark et al. 2010; Bauer et al. 2018). Most individuals living with advanced pancreatic cancer (APC) experience a rapid decline in quality of life (QOL), even when on first-line treatments (Al‐Batran et al. 2021). Addressing the high symptom burden of APC remains an ongoing challenge in disease management.

Exercise has shown not only to be a safe and effective means to improve QOL of individuals living with and beyond cancer but also shows promise in improving overall survival in this population (Baumann et al. 2024; Courneya et al. 2025). While evidence-based guidelines for exercise oncology have been developed, they are primarily based on trials with early-stage breast and prostate cancer patients (Campbell et al. 2019). Further, recent effectiveness–implementation exercise-oncology trials in the Canadian context have reflected low representation of individuals living with pancreatic cancer (Heinze et al. 2024; Wagoner et al. 2024). A scoping review of exercise training for pancreatic cancer identified a lack of evidence for the care context, specifically for individuals living with advanced disease (Rosebrock et al. 2023). Similarly, a systematic review of exercise interventions in pancreatic cancer care highlighted that exercise studies with eligibility open to individuals with all stages of pancreatic cancer (I–IV) often do not see representation from patients with advanced disease (III–IV) (Luo et al. 2021). Given the high disease burden, interventions tailored for individuals with APC and physical deterioration are needed.

Exercise oncology programs, which specifically address the unique barriers, motivators, and context-specific needs of their target, are shown to have high implementation and effectiveness outcomes (Burse et al. 2020; Molenaar et al. 2023; Wagoner et al. 2023). The Theoretical Domains Framework (TDF) is a tool to simplify and maximize the accessibility of behavior change theories (Michie 2005). The TDF includes 14 domains which serve as a robust foundation in intervention research to specify target behaviors and select study design (Cane et al. 2012). The TDF can be used in combination with the Behavior Change Wheel (BCW) to predict relevant and context-specific theoretical domains for intervention effectiveness (Cantwell et al. 2020; Grimmett et al. 2024; Langley et al. 2025). The BCW is a model used to understand the nature of behavior, determine appropriate intervention and policy strategies, and provide relevant criteria for intervention evaluation (Michie et al. 2011). Understanding the complex interactions that influence behavior is the first step of intervention development in the BCW (Michie et al. 2014). However, there is limited qualitative research exploring exercise behaviors in APC. Therefore, we aimed to develop an in-depth understanding of how individuals living with APC perceive and experience exercise in a Canadian context.

## Methods

### Study design, participants, and recruitment

We conducted an exploratory qualitative semi-structured interview study using a pragmatism worldview (Cornish and Gillespie 2009) and an interpretive description methodology (Thorne 2016). Participants were recruited from February 2025 to May 2025 after Nova Scotia Health Research Ethics Board Approval (REB File #: 1031263). We used criterion sampling to recruit individuals living with APC from across Canada. Recruitment posters were shared with cancer charity organizations in Canada, on social media, and distributed with clinician support at the Nova Scotia Cancer Center. The inclusion criteria included those who: (a) were diagnosed with stage III or IV exocrine pancreatic cancer; (b) resided in Canada; (c) were >18 years; (d) were able to communicate in English; and (e) had the capacity to provide informed consent. All interested individuals were invited to contact the research team by email or telephone, and inclusion/exclusion criteria were confirmed. Verbal informed consent was obtained from each participant prior to the interview.

### Data collection

Semi-structured interviews were conducted online through Zoom videoconference software or in-person at the Nova Scotia Cancer Centre. Interviews were conducted by C.C., a female student trainee in the Master of Science program at Dalhousie University; no other individuals were present during interviews. C.C. is an active volunteer in the pancreatic cancer community and has also lost family members to the disease. C.C. had prior interaction with 3 participants through her volunteer work and disclosed her family loss to all participants. She had previous training in qualitative interviewing through work in the Physical Activity and Cancer Lab.

The semi-structured interview guide (Supplemental File A) was developed based on the BCW and TDF (Michie et al. 2014). The guide was reviewed and revised by all authors. Specific interview questions evolved throughout each interview to probe for detail and capture in-depth understanding of experiences. Interviews were audio-recorded and transcribed verbatim by an experienced transcriptionist.

Sample size was not set *a priori* for this research study. Instead, information power was used to determine adequate sample size (i.e., a qualitative research sample size technique based on research aim, sample specificity, use of established theory, quality of dialogue, and analysis strategy) (Malterud et al. 2016). In May 2025, the research team collaboratively concluded that the richness and diversity of the data were sufficient to provide a meaningful analysis.

### Data analysis

Data were analyzed by C.C. using reflexive thematic analysis (RTA). RTA is a theoretically flexible analytic method to identify and report repeated patterns (Braun and Clarke 2006; Clarke and Braun 2017). Analysis first involved repeated active listening of interview audio recordings and reading through data transcripts. Reflexive journaling was conducted to explore possible patterns within/across the dataset and track critical engagement. After this familiarization, initial codes were produced based on potential patterns, interesting data extracts, and relationships to BCW and TDF. These codes were then manually labeled throughout the entire dataset using Microsoft Word, with 2 coding runs of each interview transcript. Next, codes were collated and sorted into broader themes. Thematic maps were used to help visually process and identify relationships among codes and themes. These candidate themes were then reviewed and refined according to congruency with both initial codes and the entire dataset. Refined themes were named and defined.

Secondary interviews (i.e., member checks) were not implemented due to limitations in advanced care, such as the rapid progression of the disease. Further, the research team identified their interpretive authority, and themes were also shared with B.P. for insight as a “critical friend” for review and discussion.

### Results

Six individuals diagnosed with pancreatic ductal adenocarcinoma were interviewed. Interviews were between 55 and 115 minutes in length. The average age of participants was 64 years (range 50–77 years), 4 participants identified as women, and all identified as White. Participants were located across Canada, and most participants had been diagnosed with stage IV disease. We identified 3 themes relating to how individuals living with APC in Canada perceived and experienced exercise: (1) *Taking control of my journey*; (2) *Building the motivation*; and (3) *If it was a program for me*. Theme descriptions and key participant quotes are presented in the following sections, and additional representative quotes are available in Supplemental File B.

### Theme 1: Taking control of my journey

This theme describes exercise in the context of the difficult and often uncontrollable journey of pancreatic cancer. Participants outlined how pancreatic cancer had become inadvertently woven into their identity and daily life:

I’m tired. I am tired of having cancer. Like I’m tired of the treatments. I’m tired of the side effects. And you don’t ever get a break. […] I look in the mirror, I’ve got cancer. I try and do things during the day. I have cancer. So it’s always, always there. And it’s exhausting. Participant 5

Participants described feeling an overall loss of control in their illness experiences due to both their high symptom burden, as well as negative health system factors. Specifically, several participants described a perceived lack of support and resources within the health system:

I just felt like I was abandoned actually. You know I mean I saw the surgeon, I got the diagnosis, and they ran a couple more tests, and then it was like, We’ll see you in a month for surgery. Well like, what do I do now? And there was nothing. Like there was no support. Participant 4

Participants emphasized wanting to make the right health decisions and actively optimize their health outcomes, often feeling that they needed to self-advocate. Motivation related to beliefs about health consequences was a key influence for exercise behavior. Exercise was discussed as a strategy to actively build hope and work toward better health outcomes:

I guess the biggest motivator is, for example, my current diagnosis is like, okay, the doctor isn’t quite sure you’ve got three months left to live. Well, if I want to prove her wrong, maybe I should get out and exercise every day. Maybe I should go for a walk every day. Participant 6

Overall, participants described wanting to take control and find hope where possible within the difficult journey of pancreatic cancer. They outlined exercise as a tool to both physically and psychologically cope with their diagnosis. They suggested that exercise could be an important supplement to their care.

### Theme 2: Building the motivation

This theme highlights challenges in maintaining motivation to exercise, often despite understanding its importance. Participants often viewed exercise in the context of their pre-cancer lifestyle, in which they had greater physical capabilities. Realistic goal setting was challenging, and the inability to meet goals was complex and emotional for participants to manage:

It’s like being on a championship team, and then you win, and then you retire, or you graduate, and you don’t have that. And you miss it. And I hate it. And I can’t do it … I hate the fact I can’t do what I used to do five years ago. Participant 2

Participants also described feeling that exercise was not one of their key priorities, especially when they were already feeling overwhelmed by their diagnosis:

I’m sorry when your oncologist sits down and says, ‘You know what, we think you have a year to live.’ I mean it’s like, Oh my god … Like what am I gonna do? It’s just you know, it’s like, Oh … I just, you know, exercise was, at that point, exercise was the farthest thing from my mind while I was in his office. Participant 4

Participants felt that they had limited energy and that they needed to prioritize their time for their day-to-day activities. They described hesitancy in joining an exercise program due to uncertainty of what the program might entail and if it would be worth their time. For exercise programming to be appealing, participants detailed that it would need to fit in their existing schedule and routines:

I haven’t had the energy and I just … I’m still busy. I’m cooking during the day, or shovelling snow. I’m running around doing shopping … So I’m running around a lot. So I’m still doing everything I did before I had cancer. Participant 3

Participants described that after beginning an exercise program, tracking emotional and physical outcomes of initial exercise efforts could help in continued motivation. They described graded tasks and programming with low initial commitment as strategies to help build motivation:

And so, once you get started on it … And it should be simple and easy. Simple to see, no big instructions, and easy to do. And then just keep encouraging you. It’s like, Oh yeah, okay, that feels good. I feel that. I know that that is good. And there could be an option for, you know, okay, if you want to do level two, here’s another one. Or if you can add that, you know. But the main thing is to get people started. Participant 6

Participants also described that exercise could become more of a priority if it was considered part of treatment and endorsed by their healthcare providers:

But it would be pretty great if it was kind of a requirement for your treatment. Especially when the research says it’s all good. Like this is, you know, all good. And if it was tailored to your particular cancer, it would be kind of a therapy. You know, exercise as a physical and mental healing, you know, kind of. If it was taken as seriously as chemo, that would be pretty great, you know. Participant 5

Overall, participants described having limited time and many priorities to balance in their cancer journey. Although exercise was not currently a main priority for participants, they suggested that setting realistic goals and reframing exercise as treatment could help in their motivation.

### Theme 3: If it was a program for me

This theme describes how participants felt that they would exercise *if* there was a program available which was tailored for their needs. Participants described a lack of opportunity to engage in exercise in terms of not knowing that exercise oncology supports were available. They felt that they would join an exercise program if these barriers were mitigated and exercise was included as part of their treatment:

I did ask my doctor, Can you write a prescription for the gym? Can you write a prescription for the walking track? And she said, Like I can’t. It’s not an option. So it just feels like there’s a lot of barriers. Participant 5

Participants described the transition between diagnosis and treatment as a specifically stressful time when they were looking for hope and support from the health system. Participants suggested that an exercise program may have been beneficial during this time to provide that support:

I’m thinking now of the month I had between basically the last time I saw the surgeon, prior to the surgery. I think at that point it would have been really nice to have some sort of guidance, because I didn’t know what to do. I mean I ended up reupholstering my furniture because I had to do something, you know. So that probably would have given me something to do and something to focus on. Participant 4

Participants felt that it would be important to have programming specific to their stage of health. They described potential benefit of an exercise program that was modified for pancreatic cancer care and their individual needs:

And to get on like a program that I know like is … You know, to kind of take the guesswork out of it, for me, I guess. Like that am I doing enough? Am I not? And then to be in a program that is tailored toward that, then I would know that it is right, it is enough. Participant 1

Participants identified that a supportive instructor would help build motivation to participate in exercise programming. They explained the importance of positivity, joy, and support within an exercise program:

Because that’s the perception of who we are, and how long we’re going to last, you know. So, yes, the opportunities are all there. If I want to Google, you know, an exercise program, I can totally do that. But for me, I want it to be, you know, like I said, vetted. I want it to be one that’s for people like me, and safe for people like me. Participant 5

Overall, participants described the importance of a program that was tailored for progressive functional decline, various levels of commitment, and their individual needs. They felt that a program would have to be readily available and a good fit for them to consider joining.

## Discussion

This study provides insight into the experiences and perceptions of exercise among individuals living with APC in Canada. Participants described exercise as a tool to gain control in the often-uncontrollable journey of pancreatic cancer. They voiced the desire to be more active, while emphasizing considerable challenges in building motivation to exercise. Participants perceived that an exercise program integrated with their cancer treatment, which included progressive programming and a supportive instructor, could help increase their level of physical activity.

Exercise was highlighted by participants in this study as a means of gaining control over their mental and physical health. Perceived control, a concept describing the perception that events are contingent on one’s behavior, has been described in the literature as an important component of behavioral reinforcement (Rotter 1966). Reclaiming control of cancer-related symptoms has been shown to be a key motivating factor for exercise engagement in advanced cancer and is an important facet of QOL and hope in the illness experience (Shukla and Rishi 2018; Bland et al. 2022; Young et al. 2024). Hope and control should be explored as outcomes for APC exercise research.

Participants in this study described experiencing high symptom burden and reduced physical capability, ultimately leading to low self-efficacy and motivation to exercise. Conceptual modeling of the APC illness experience has identified reduced physical function as a detrimental outcome with high spillover effect on social activities, ability to work, and emotional wellness (Herman et al. 2019). Participants in our study outlined the spillover effect of poor physical function on their motivation to exercise. Education and modeling strategies to reframe preconceived notions of exercise may be beneficial to improving beliefs about capabilities and motivation to exercise. Additional strategies, such as motivational interviewing, clinician assessment and referral, and the involvement of knowledgeable and supportive instructors, may also play a vital role in enhancing motivation by strengthening positive beliefs about the benefits of exercise and increasing confidence in ability to engage in exercise (Harkin et al. 2023; Cormie et al. 2025).

Exercise programming for APC care should also explore progressive programming with graded commitment and tasks. Participants in our study described hesitancy to join an exercise program due to the large perceived commitment and many competing priorities. Most advanced cancer exercise oncology programs in the literature include at least 2 sessions a week, with an average session duration of 60 minutes, and program length of 8–12 weeks (Rodríguez‐Cañamero et al. 2022). Stepped-up exercise oncology programs, where initial program commitment is low and builds with participant request, should be further explored in the APC care context.

Time toxicity is an emerging concept in oncology literature characterizing time patients spend in coordinating care and pursuing cancer-directed therapy (Gupta et al. 2022). The concept outlines the impact of meaningful time lost due to pursuing intensive cancer treatment with potentially only modest survival benefit (Gupta et al. 2024). Participants in our study described uncertainty about what an exercise program for APC care might entail, and if it would be worth their time. Integrating exercise programming in APC care would necessitate careful consideration of expected benefits compared to potentially valuable time lost with family and friends. Strategies to optimize protocol logistics (e.g., virtual appointments to avoid transportation challenges) may be considered to reduce time toxicity (Dona et al. 2024).

Exercise intervention in pancreatic cancer care has potential for high *value*, a multidimensional concept linking cost of an intervention and impact on patient outcomes (Goulart 2016). There has been recent movement for oncology to recalibrate toward a more patient-centered approach with prioritization of treatments which improve survival *and* QOL (Booth et al. 2023). In the context of APC care with limited treatment options, exercise training provides a potentially valuable intersection of physical function maintenance, psychological support, and palliative care. Tailored exercise programming for APC care that incorporates evidence-based behavior change techniques should be considered.

### Strengths and limitations

This qualitative study provides an in-depth understanding of patient perceptions of exercise in APC care. This knowledge contributes to exercise oncology literature by providing analysis of exercise experiences in the context of the BCW and TDF. Future work can incorporate findings from this study to tailor exercise programming for APC care.

This study was exploratory and does not aim to generalize findings to all contexts. Indeed, this study comprised a small sample, and there is inherent variability in experiences and perceptions between participants. The notion of inferential transferability better describes the purpose of this work to provide a thick description of experiences whereby the reader can assess their transferability to another context (Lewis and Ritchie 2014). However, lack of diversity among participants is a limitation of this work. All participants identified as White, and thus intersecting considerations with race cannot be explored. It is also likely that there was selection bias in that participants in this study had more interest in exercise and/or research compared to most individuals living with APC.

### Implications to practice

The information provided from these interviews provides helpful considerations for program tailoring. Based on the results, recruitment materials for exercise programming within APC care should highlight specific cancer-related benefits of exercise. Including testimonials from individuals living with APC may also help individuals develop belief in their capabilities. Clear details of what programming entails should be provided, and time toxicity should be reduced where possible. Options for programming which have limited time commitment should be explored, and progressive commitment opportunities should be offered. The integration of motivational interviewing strategies, clinician support, and trained exercise professionals should be explored to further enhance motivation. Further research should be conducted to assess the effectiveness of exercise programming to improve QOL and survival in APC.

## Conclusion

Exercise was perceived by individuals living with APC as a way to gain control of their physical and mental health. This study outlines the importance of building motivation to exercise through progressive programming and graded tasks. Exercise programs in the APC care context should aim to maximize value by creating a supportive environment and minimizing time toxicity where possible. Overall, exercise programming in APC care could be a valuable supplement to treatment of individuals living with an often-uncontrollable disease.

## Supporting information

10.1017/S1478951526103356.sm001 Cyr et al. supplementary material 1 Cyr et al. supplementary material

10.1017/S1478951526103356.sm002 Cyr et al. supplementary material 2 Cyr et al. supplementary material

10.1017/S1478951526103356.sm003 Cyr et al. supplementary material 3 Cyr et al. supplementary material

## Supplementary material

The supplementary material for this article can be found at https://doi.org/10.1017/S1478951526103356.
