# Supplementary material for: Exploring patient perspectives of exercise in advanced pancreatic cancer care in Canada: A qualitative interpretive description
Source: Palliat Support Care. 2026 Sep 4;24:e214. doi: 10.1017/S1478951526103356 (PMC13735770; doi:10.1017/S1478951526103356)
Supplement: Supplementary file 2 [file S1478951526103356sup002.docx]

**Supplemental File A.**

**Interview Guide**

Exploring patient perspectives of exercise in advanced pancreatic cancer care in Canada: a qualitative interpretive description

Chloe Cyr^1,2,3,4^, Ben Perry^3^, Ravi Ramjeesingh^3,5^, Chris M. Blanchard^1^, Melanie R. Keats^1,2,4,6^

1. Faculty of Medicine, Dalhousie University, Halifax, Nova Scotia, Canada
2. Physical Activity and Cancer Lab, Halifax, Nova Scotia, Canada
3. Craig’s Cause Pancreatic Cancer Society, Canada
4. Beatrice Hunter Cancer Research Institute, Halifax, Nova Scotia, Canada.
5. Department of Medicine, Division of Medical Oncology, Dalhousie University, Halifax, Nova Scotia, Canada.
6. School of Health & Human Performance, Faculty of Health, Dalhousie University, Halifax, Nova Scotia, Canada

Corresponding Author: Dr. Melanie Keats

Email: melanie.keats@dal.ca

Semi-Structured Interview Guide

Tailoring Exercise for Advanced Pancreatic Cancer Care: Understanding and Integrating the

Patient Perspective

Introduction:

Hello, [participant name]. I want to begin by thanking you for speaking with me today. I am

very happy to connect with you and learn more about your journey with pancreatic cancer.

My name is Chloe, and I am a student researcher at Dalhousie University. I am also a

research assistant with Nova Scotia Health. I study pancreatic cancer and through family

experience, I have seen how difficult this disease can be to manage. It is my goal to help

improve pancreatic cancer care in Canada.

Today, I am hoping to learn more about how you feel about exercise as a component of

cancer care. I am very interested to explore your perspectives, opinions, and ideas. I want

to remind you that your participation in this study and this interview is voluntary. We can

stop or pause at any time. You can also choose to skip any questions that you may not want

to answer.

Do you have any questions?

May I start the recorder?

[Begin recording]

Before we begin the interview, can you please confirm that you have reviewed all the

information in the consent form related to this study?

Can you please explain briefly, in your own words, the purpose of this study?

Have you been given opportunity to discuss this study and have all your questions been

answered to your satisfaction?

Do you agree for this interview to be recorded, as described in the consent form?

[If no, thank the participant for their time and end the session]

Do you understand that participating in the study is completely voluntary, and you can

withdraw at any time?

Do you understand that you do not have to answer every question, and if you find a

question too hard, we can skip it?

Would you like to continue?

Thank you.

[Document verbal informed consent]

Section 1: Context and Cancer Journey

To start, I’d like to ask some general questions about your journey with pancreatic cancer:

1. When were you diagnosed with pancreatic cancer?

Prompt: do you remember the month and year that you were diagnosed?

2. What type? What stage of pancreatic cancer are you living with now?

Prompt: pancreatic cancer can be either exocrine or endocrine. Exocrine pancreatic cancers include ductal adenocarcinomas, acinar cell carcinomas, and squamous carcinoma. Endocrine pancreatic cancers are often referred to as neuroendocrine tumors.

Prompt: staging is a way to classify cancers. For example, Stage III is when pancreatic cancer has spread to several nearby lymph nodes or is growing near major blood vessels. Stage IV is when the cancer has spread from the pancreas to other organs, like the liver or lungs.

[Confirm eligibility criteria – exocrine pancreatic cancer; stage II or IV/locally advanced or

metastatic]

3. What treatments have you received so far for your pancreatic cancer?

Prompt: Have you had surgery, chemotherapy, radiation, or immunotherapy?

Prompt: Have you had supportive care such as pancreatic enzyme replacement therapy, nutritional counselling, psychosocial counselling, or exercise counselling?

4. What do you find to be the most distressing symptom relating to your pancreatic

cancer or treatments?

Prompt: symptoms can include physical, psychological, social, financial, or spiritual challenges.

5. How has your ability to perform everyday physical tasks changed since being

diagnosed with pancreatic cancer?

6. How has your quality of life changed since being diagnosed with pancreatic cancer?

Prompt: quality of life is a broad term and can include aspects of your mental health, physical health, social relationships, environment, spirituality, status, wealth, sense of security and safety, and belonging.

7. What challenges do you experience in maintaining your quality of life?

a) How do you cope with or overcome these challenges?

Section 2: Exercise Experiences and Perspectives

Thank you for sharing. I’d like to ask now about your personal experiences with exercise.

8. Exercise can mean different things to different people. What does exercise mean to

you?

a) What images or experiences come to mind when I say the word “exercise”?

b) Can you tell me what you think that exercise entails?

In research, exercise is typically as a specific type of physical activity done to improve or

maintain fitness. Physical activity is then a broader term describing all movement

throughout the day. Sedentary time is a related concept describing time spent sitting,

reclining or lying down.

Do these definitions for research make sense to you?

[Confirm]

9. Thinking back to before your pancreatic cancer diagnosis, how would you describe

your level of physical activity? Exercise? Sedentary time?

10. Thinking now to the present, how would you describe your level of physical activity?

Exercise? Sedentary time?

11. What skills and capacities do you think are required to exercise?

a) How confident do you feel in your ability to engage in exercise, and what

factors contribute to that level of confidence?

b) Do you believe that you can incorporate exercise into your current daily

routine? Why or why not?

Prompt: Do you think that you have the resources, skills, and time?

12. In your daily life, how do you decide if you are going to engage in exercise?

13. How have your goals relating to exercise changed since being diagnosed with

pancreatic cancer?

14. Are your friends and family physically active?

a) How do you feel that their activity influences your level of activity?

15. What knowledge do you have regarding the benefits of exercise during cancer

treatment?

a) How do you perceive the relationship between physical activity and managing your symptoms or treatment side effects?

16. What is your understanding about exercise guidelines for people living with

pancreatic cancer?

Prompt: what intensity, duration, and types of activities do you think are

recommended?

17. If you wanted more information about exercise, where would you go?

Prompt: who would you ask?

Section 3: Exercise Intervention Barriers, Facilitators, and Recommendations

Thank you. In this next section, I’m now hoping to learn more about your ideas about

exercise as a supportive resource in pancreatic cancer care.

Imagine that after this interview, I tell you that you can join an exercise program designed

for people living with and beyond cancer, at no cost.

18. Do you think that you would join the exercise program?

a) Why or why not?

b) How might your decision change if you knew that the program was

specifically designed for people living with advanced pancreatic cancer?

19. If you did join the exercise program, what challenges do you think you might

experience?

a) To what extent do you think that you could manage or address these

challenges?

b) What strategies could you use to address or overcome these challenges?

Prompt: How might healthcare providers, family, friends, support groups, or exercise professionals play a role?

20. What benefits do you think you might experience?

a) How important are these benefits to you?

21. In your opinion, what would be the best way for exercise to be integrated into advanced pancreatic cancer care?

Section 4: Demographics

I want to sincerely thank you for sharing your experiences with me today. I am now going to

ask a few demographic questions.

22. What is your current age?

23. What is your gender identity?

Prompt: woman, man, non-binary, etc.

24. What is your sex?

Prompt: male, female, intersex.

25. What is your race?

Prompt: White, Black, Asian, Hispanic, etc.

26. What is your marital status?

Prompt: married, never married, living common law, widowed, divorced,

separated, etc.

27. What is the highest level of education that you have you received?

Prompt: high school, community college, trade or technical school,

bachelor’s degree, graduate degree, etc.

28. Do you live in a rural or urban location?

29. Do you consider yourself to be financial stable?

30. How do you feel that your demographic identities impact your experiences with

exercise and pancreatic cancer?

Section 5: General

31. Before we conclude this interview, is there anything else relating to your experiences

with pancreatic cancer and exercise that you would like to add?

32. Do you think that we missed any important points or topics today?

Conclusion:

Thank you for your time today. This is the end of the interview. I appreciate your willingness

to share and contribute to this research.

Thank you.

[End Recording]
