# Supplementary material for: Exploring patient perspectives of exercise in advanced pancreatic cancer care in Canada: A qualitative interpretive description
Source: Palliat Support Care. 2026 Sep 4;24:e214. doi: 10.1017/S1478951526103356 (PMC13735770; doi:10.1017/S1478951526103356)
Supplement: Supplementary file 3 [file S1478951526103356sup003.docx]

**Supplemental File B.**

**Representative Quotes**

Exploring patient perspectives of exercise in advanced pancreatic cancer care in Canada: a qualitative interpretive description

Chloe Cyr^1,2,3,4^, Ben Perry^3^, Ravi Ramjeesingh^3,5^, Chris M. Blanchard^1^, Melanie R. Keats^1,2,4,6^

1. Faculty of Medicine, Dalhousie University, Halifax, Nova Scotia, Canada
2. Physical Activity and Cancer Lab, Halifax, Nova Scotia, Canada
3. Craig’s Cause Pancreatic Cancer Society, Canada
4. Beatrice Hunter Cancer Research Institute, Halifax, Nova Scotia, Canada.
5. Department of Medicine, Division of Medical Oncology, Dalhousie University, Halifax, Nova Scotia, Canada.
6. School of Health & Human Performance, Faculty of Health, Dalhousie University, Halifax, Nova Scotia, Canada

Corresponding Author: Dr. Melanie Keats

Email: melanie.keats@dal.ca

Table 1. Additional representative participant quotes by theme

| **Theme** | **Quote** |
| --- | --- |
| Taking control of my journey | “Because like every two weeks, you know…like it’s like that impending doom, right. Because you're, Oh God, I'm just feeling good, and then I'm going to get another round, and then it's going to make me feel bad again.” P1  “I like doing it because I feel so good afterwards. And I also find that it really calms me down. There’s just some days that I just feel so stressed. And if I go for a walk, or I go to the gym, and I come back home, then I feel I can deal with whatever it is that was causing me the stress. So I know the benefits that are there, that's why I try to do it.” P4  “And so your life kind of…not closes in, but you kind of focus more on your health and what you need to do, and everything that's possible to make this…you know, to have a positive outcome.” P6  “You know, the statistics are so poor. And that you're looking and trying to grab a hold of any kind of hope whatsoever that something is going to work for you.” P1  “So, you know, I mean, yes, you feel better and all the rest of that kind of stuff. But for me, what would be even more helpful, more motivating is the physical benefit. What's the physical thing that happens with cancer when I do some exercise?” P6  “There’s delays with getting treatment. You know, diagnosis and treatment. So in that timeframe, that's a very, very stressful, stressful time. That the patient feels like they've been diagnosed, and then they get left out to hang and to figure things out for themselves.” P1  “And sort of the way he reacted, I actually didn't go back. Because I kind of thought I need to surround myself with people that are going to believe in my wellness, whatever form that takes.” P5  “I have a very good friend whose mother died of pancreatic cancer 25 years ago, and it's almost as if nothing has changed. And I just, I can't believe that. Like I just can't believe that nothing has changed. I can't believe that they don't know any more than they did then. Like I just can't believe that.” P4  “I guess I wish there was more things they could do for it at the present time. […] You know, you go the grocery store shopping, or the mall, and you see people standing there with little tables - breast cancer, lung cancer, this and that. I never see nothing for pancreatic cancer.” P3  “I was disappointed in the [city] because they have that awesome heart program, like I mentioned earlier, the heart program. They don't have anything for cancer patients. And that's really a shame because I think a lot of us have it, and a lot of us would take advantage of the exercise, like the coaching and the monitoring, and, you know, just the benefits of just being in a safe space with other patients who are going through something.’ P5  “I'm thinking now of the month I had between basically the last time I saw the surgeon, prior to the surgery. I think at that point it would have been really nice to have some sort of guidance, because I didn't know what to do. I mean I ended up reupholstering my furniture because I had to do something, you know. So that probably would have given me something to do and something to focus on.” P4 |
| Building the Motivation | “And I'm still experiencing tiredness from last week, you know, the chemo event. It's about nine days of tiredness out of a two week cycle. So it was like, you know, you can't do anything”. P6  “Yes, because I'm doing a lot of things to balance my life, to keep a balance. They're all very involved in maintaining health. You know, eating properly, resting properly, you know, getting outside, shopping for the proper groceries, planting in my garden. So, you know, like I have a lot of things that I do, that this would have to fit in. To put it that way, yeah.” P6  “But then having family or a friend just…or like to call me up and say, Hey, what’s going on? You know, Let's do this walk, or, Let's do this hike, or whatever, it's like, Yeah, okay. That's better. Better than me making the decision to do it myself.” P1  “In fact, you can’t get out and walk, you know, or do any exercise because you’re dizzy. You’re absolutely unbelievably dizzy.” P6  “Exercise to me is, yeah, you’ve got to pretty much bring yourself close to death and then bring yourself back again. [laughs] You’ve got to work out hard in order to get the benefit from that. I don’t… Yeah, I was never like somebody that believed in like light exercise. I was always one that always went like full on.” P1  “Well, I always think people love this. Like people love doing this. I don’t love it. I don’t even like it. […] And I’m like, You go, because I don’t care. You know, I would love to. You know, if I could instantly be that person, I would choose to be that person. Again, that’s terrible because I could choose to be that person now. But if I could like snap my fingers and become a runner, and it would be awesome, yeah. But it’s really hard. I just… Yeah, I’m not… I’m a couch-y. I’m not a run-y.” P5  “Yeah, very hesitant. And it's a bit of like an emotional struggle. Again, like the anxiety, right. Because I know that I need to exercise, and I want to exercise, but I just… You know, there's a little bit of pain with that, and just the fear of making it worse.” P1  “I would be interested in like attending the first section to see you where it would go. I'm always willing to learn.” P4  “Well, I think, Chloe, if you were to ask me, I said I would do it. But I hate being disappointed. Like, you know, let's say they say, Well, we want you to go out and walk 10 minutes a day. And it's a day here that's raining, and I don't want to do it. Then I'm disappointed, right. And like the people in the study are like, Oh, this guy's not doing it. But they’ve got to keep in mind, I’ve had cancer for five years, pancreatic cancer. Like I'm lucky to be here. Like getting off the couch for 10 minutes is huge. It's huge. It's hard, you know. So if I could do something where there was no expectations. Did you do the workout? Yes, no. How did it feel? Whatever. You know, didn't do it today. Because if there's something like, I don't know, I would think it'd be three, four times a week, maybe it's every day, because it's not a lot. If it's every day, it's not going to happen, right, because I'm busy, too, right. Not that busy. But like the weekend was very busy.” P2  “Well, this would be neat out of your program, where it's like, okay, this is recommended for those with cancer who might be suffering these kinds of things, try this little five minute thing. And if you feel good, here, add this five minutes to it. Just do it once a day. Something easy.” P6 |
| If it were a program for me | “I don't think I’d want to be going back to the gym. It's just too much. I don't want to spend the money, to be honest with you.” P3  “Like if I had to drive, like say like come into the city here, you know, every day to do it, I'm not sure if I would. Because if it was like a stormy day or whatever. […] So if I had to come into a facility, I probably wouldn't do it as often.” P1  “Like when I go out, I have to wear a mask. Wearing a mask at the gym is hard. You know, that's not, you know, a reasonable expectation. And so the fear of germs gets in there, too.” P5  “But there really doesn't feel like there's either an availability or an awareness that there's exercise for cancer patients, or it's not well advertised, I'll say. You know, it's not well… I mean I've used the bathrooms. I've been in the cancer centre hallway. You know, I go up to chemo. There's lots of areas where things are posted, and, you know, you can join these groups, or you can… The wellness, you know, or the beauty wellness and stuff like that. But, you know, really, now that you've asked the question and I'm thinking about it, I don't know that there's sort of…you know, an exercise therapist that would be available just for cancer patients.” P5  “Yeah, like finding stuff that would work for various stages of health. Like mine, health is terrible. You know, like I'm thinner and weaker. I like being a little thinner, but not this thin. I wish I was like 10 pounds more. But, you know, so somebody that's thin, weak, can't do strenuous stuff. And then you get guys like just started, you know, still got lots of energy. They don't have the same energy, but they don't know that yet. So you give them like a full workout. You know, like various stages I think would be good. Like meeting the needs of various athletic abilities of pancreatic cancer patients, I think would be good.” P2  “I mean the personal contact is really helpful. […] So a call from an exercise specialist, but that had…that was… The call was more involved in a conversation with the person about their level of exercise. What do you find about it? Does it work for you? What has worked for you, kind of thing.” P6  “You know, there would be just acceptance of where you're at and what you can do. And there wouldn't be any… And there'd be a joy in that. There'd be a joy in and celebration in just being able to do what you can on that day. You know, there's no… Like there wouldn't be, Well, you did 50 push-ups the other day, how come you can only do three? You know, kind of thing. There'd be just that celebration of like, Hey, you're here.” P5  “And you have sort of a camaraderie or a kinship with that. And with cancer, it's just it's very isolating.” P5 |
